# Evaluation of nasal epithelium sampling as a tool in the preclinical development of siRNA-based therapeutics for asthma

**DOI:** 10.1111/jcmm.12014

**Published:** 2013-02-13

**Authors:** Gareth D Healey, Neil Evans, Julian M Hopkin, Gwyneth Davies, William Walker

**Affiliations:** College of Medicine, Institute of Life Science, Swansea UniversitySwansea, UK

**Keywords:** Asthma, biomarker, CCL26, nasal epithelium, preclinical, siRNA, STAT6

## Abstract

The development of siRNA-based asthma therapeutics is currently hampered by a paucity of relevant biomarkers and the need to ascertain tissue-specific gene targeting in the context of active disease. Epithelial STAT6 expression is fundamental to asthma pathogenesis in which inflammatory changes are found throughout the respiratory tract. Therefore, to improve preclinical evaluation, we tested the efficacy of STAT6-targeting siRNA within nasal epithelial cells (NEC's) obtained from asthmatic and non-asthmatic donors. STAT6 expression was invariant in both donor groups and amenable to suppression by siRNA treatment. In addition, STAT6 mRNA was also suppressible by apically delivered siRNA treatment in comparative differentiated nasal epithelial cell-line monolayer cultures. Analysis of donor NEC's showed consistent elevation in CCL26 (eotaxin-3) mRNA within the asthmatic group suggesting potential as a relevant biomarker. Furthermore, targeting of STAT6 with siRNA attenuated IL-13-driven CCL26 expression in these cells, pointing to the utility of this approach in preclinical testing. Finally, siRNA-mediated suppression of STAT6 was independent of donor disease phenotype or epithelial cell differentiation status, signifying therapeutic potential.

## Introduction

Asthma is a complex inflammatory disorder in which interleukin (IL)-13 acting through STAT6 has been identified as a major driver of bronchial inflammation [1–3], prompting efforts to therapeutically target this pathway [4–6]. STAT6 expression in lung epithelial cells is exclusively required for IL-13-mediated pathology [7], consistent with the increasing recognition that epithelial cells play a fundamental role in asthma pathogenesis [8, 9]. Against this backdrop, and given the mainstay of asthma therapy continues to rely on the use of inhaled steroids [10], which can have undesired side effects [11], we have developed a more refined therapeutic approach that harnesses the endogenous RNA-interference mechanism to specifically suppress STAT6 expression in epithelial cells using small interfering RNA (siRNA) [12, 13]. However, limitations associated with animal models of allergic asthma [14, 15], a paucity of relevant biomarkers [16, 17] and with particular regard to siRNA-based treatment—the status of target gene expression within the respiratory epithelium of asthma sufferers—mean that the drug-development pathway for new asthma therapeutics can be particularly problematic. To address these issues we postulated that nasal epithelial cells (NEC's) from asthmatic donors would lend themselves to more appropriate preclinical testing of candidate siRNA. This premise was based on substantial evidence that respiratory allergy is an integrated systemic disorder [18, 19], in which similar inflammatory events have been found in the upper (nasal) and lower (bronchial) airways, leading to the increased recognition that conditions such as asthma should be treated as a single airway disease [20]. Evaluation of this approach was also warranted given that techniques involved in nasal sampling are relatively simple and non-invasive compared with the more traditional lung sampling methodologies.

To advance preclinical development we extend our own work [12, 13] and previous studies on nasal epithelial sampling [21, 22]. By assessing target gene expression and the ability of siRNA to suppress this expression in NEC's obtained from asthmatic donors, our aim was to evaluate the usefulness of primary NEC's as a model for siRNA-based asthma drug development. As a further reference for these studies we performed similar analyses in a well-characterized model of differentiated human nasal epithelium which employs Roswell Park Memorial Institute (RPMI) 2650 cells grown as air–liquid interface (ALI) monolayer cultures, previously shown to be a suitable model for the *in vitro* screening of nasal drug candidates [21, 23]. Given that *ex vivo* drug treatment would require a time window for siRNA-mediated suppression to operate [12], in addition to analysing expression of our target gene (STAT6), we also analysed expression of a representative panel of genes linked to both disease (asthma) phenotype and epithelial differentiation status in both nasal models. We included CCL26 (eotaxin-3) and CHI3L1 (chitinase-3-like-1) expression in this panel, as in addition to their involvement in asthma pathogenesis [24, 25], their expression is also linked to epithelial differentiation [26, 27], with both being members of gene families identified as potential asthma biomarkers [28]. CCL26 expression was of particular interest as we have previously shown this to be the predominant eotaxin family member expressed in lung epithelial cells following IL-13 stimulation, [12] and it's STAT6-dependent regulation provided a functional measurement of target gene inhibition. Expression of mucin (MUC5AC) and prominin (PROM1) gene family members associated with mucociliary differentiation [29] was also monitored for comparative purposes.

## Materials and Methods

### Culture of human NEC's

#### Primary NEC's

Asthma was confirmed by respiratory physician and sub-classified into mild, moderate or severe asthma using GINA guidelines [30, 31]. For this pilot study, numbers of asthma donors = 19 in total (mild asthma = 5, moderate asthma = 4, severe asthma = 10) and healthy non-asthma controls = 4. Exclusion criteria were presence of rhinitis symptoms, nasal corticosteroid use in preceding 4 weeks, upper respiratory tract infection in preceding 6 weeks or history of smoking, patient details and baseline data for mild, moderate and severe asthma patients are provided in Table 1. All participants provided informed written consent, with study approval from the South West Wales ethics committee. Healthy non-asthmatic volunteer controls had no history of atopy, asthma, rhinitis or smoking. Primary cells were isolated from nasal passages using the Rhino-Probe™ nasal curette (JB Morphet Ltd, Ipswich, UK). Curette samples were divided into two equal aliquots—one for time-of-sampling gene expression analysis, the other for culture/siRNA treatment. To promote normal polarized growth, cells were seeded onto collagen-coated 6-well tissue culture plates (1 well/sample) in 2 ml complete Bronchial Epithelial Growth Medium (BEGM), supplemented with epithelial growth factor, retinoic acid (Lonza, Slough, UK), 2% Ultroser G (Pall, Saint-Germain-en-Laye, France) gentamicin and amphotericin-B.

**Table 1:** Baseline data for mild, moderate and severe asthmatic patients. Asthma severity was defined by GINA (Global Strategy for Asthma Management and Prevention) guidelines. Positive atopic status was defined as a positive radioabsorbent test to 1 of 6 common aeroallergens. FEV1: forced expiratory flow in 1 second; NA: data not available. Healthy (non-asthmatic) volunteers were between 26 and 52 years of age, of white ethnic origin and had no history of asthma, atopy or smoking

Sex	Age	White ethnic group	Asthma severity	% Predicted FEV1	Atopy	Oral cortical steroids (OCS)	Daily dose of OCS (μg)	Inhaled cortical steroids (ICS)	Daily dose of ICS (μg)	Omalizumab therapy
F	38	Yes	Severe	79.6	Yes	No	–	Yes	2000	Yes
F	39	Yes	Severe	72.8	Yes	No	–	Yes	2000	No
F	51	Yes	Severe	43.7	No	Yes	17	Yes	2000	No
F	27	Yes	Severe	79.8	No	Yes	40	Yes	2000	No
F	19	No	Severe	70.7	Yes	No	–	Yes	1600	No
F	47	Yes	Severe	75.2	Yes	No	–	Yes	2000	Yes
M	40	Yes	Severe	67.8	Yes	No	–	Yes	1600	No
F	40	Yes	Severe	48.8	Yes	Yes	20	Yes	2000	No
F	35	Yes	Severe	78.8	Yes	No	–	Yes	2000	Yes
F	46	Yes	Severe	68.8	No	No	–	Yes	2000	Yes
M	51	Yes	Moderate	77.8	Yes	No	–	Yes	800	No
M	45	Yes	Moderate	76.7	Yes	No	–	Yes	2000	No
F	43	Yes	Moderate	83.2	Yes	No	–	Yes	800	No
F	59	Yes	Moderate	80	No	No	–	Yes	2000	No
M	49	Yes	Mild	74	Yes	No	–	No	–	No
F	47	Yes	Mild	103	Yes	No	–	Yes	400	No
F	NA	Yes	Mild	NA	Yes	No	–	No	–	No
M	41	Yes	Mild	105	Yes	No	–	No	–	No
M	41	Yes	Mild	88	Yes	No	–	No	–	No

#### ALI cultures

RPMI 2650 cells were routinely maintained in minimal essential medium (αMEM) as recommended (ECACC, Salisbury, UK). For ALI cultures, cells were seeded onto 0.4 μm collagen-coated Transwell™ support membranes (Sigma-Aldrich, Dorset, UK). ALI culture medium consisted of αMEM containing 2% Ultroser G, gentamicin and amphotericin-B. Once confluent, apical medium was removed and the medium volume in the baso-lateral compartment reduced to a minimum to prevent leakage. In certain ALI experiments 5 ng/ml of IL-13 (PeproTech EC, London, UK) was added daily to the baso-lateral compartment of confluent, differentiated (≥day 20) cell monolayers.

### Small interfering (si) RNA treatments

STAT6-specific siRNA (si372) was synthesized using standard chemistry and annealed by Agilent Technologies, Inc. (Delaware, USA). Scrambled control siRNA was *Silencer*^*®*^ Negative Control #1 (Life Technologies, Paisley, UK). ALI-cultured and primary epithelial cells were transfected with siRNA using the PEI-based transfection agent Interferin™ (PolyPlus-transfection Inc., NY, USA). In certain siRNA experiments, cells were cultured in the presence of IL-13 (50 ng/ml) for the last 24 hrs of culture. Immediately prior to transfection of ALI cultures, medium from the baso-lateral compartment was removed and the bottom of the Transwell™ insert sealed with sterile adhesive film. Transfection at the apical surface was then performed in 1 ml of BEGM containing 2% Ultroser G. After 24 hrs, BEGM was removed from the apical compartment, sealing film removed and sufficient fresh BEGM returned to the baso-lateral compartment to maintain the ALI culture.

### Gene expression analysis

Total RNA was isolated using Tri-Reagent® (Sigma-Aldrich) and real-time RT-PCR performed with TaqMan gene expression assays: STAT6 Hs00598625_m1; CCL26 Hs00171146_m1; CHI3L1 Hs00609691_m1; MUC5AC Hs00873651_mH; PROM1 Hs01009257_m1; GAPDH Hs99999905_m1; β-actin Hs99999903_m1 (Life Technologies). The expression of each test gene was normalized against expression of the housekeeping genes, GAPDH & β-actin (which were invariable in their expression between different donor groups and treatments). For STAT6 mRNA analysis, quantification was performed with a standard curve of recombinant human STAT6 and results expressed as percentage STAT6 knockdown compared with a transfection reagent (TF) only (negative) control. For other genes, expression was compared with relative expression in the presence of transfection reagent only (fold change) using the method described by Pfaffl *et al*. [32]. Per cent mRNA remaining was calculated by multiplying the fold- change value obtained by 100. Per cent inhibition was calculated using the following formula: % inhibition = 100 × (one−fold change).

### Statistical and data analysis

Data from multiple experiments are expressed as mean ± SD. For mRNA fold-change data, differences between groups were examined for statistical significance using the Mann–Whitney *U*-test. Quantitative measurements were compared using Student's *t*-test. In all cases, *P* < 0.05 was considered significant.

## Results

### Evaluation of nasal epithelial sampling in human asthma

**Fig. 1:**
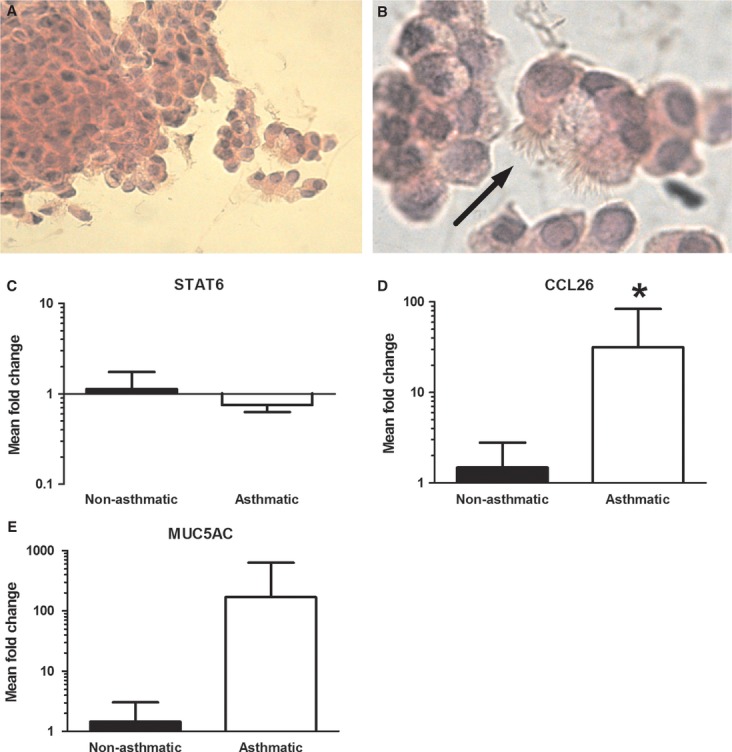
Representative micrographs stained with haematoxylin & eosin to show (**A**) sheets of differentiated columnar epithelium obtained by Rhinoprobe™ curettage sampling (40×), and (**B**) the presence of beating cilia (indicated by arrow) which were readily discernible upon placement in culture (100×). RT-PCR analysis of NEC's was carried out at the time of sampling to compare (**C**) STAT6, (**D**) CCL26 and (**E**) MUC5AC mRNA expression between non-asthmatic (closed bars) and asthmatic (open bars) donor groups. Values presented are the mean fold-change, relative to the mean δCt value for the non-asthmatic group ± SD (non-asthmatic control: *n* = 4, asthmatic donors: *n* = 19). Data were analysed by Mann–Whitney *U*-test and values differ from non-asthmatic: *P < 0.05.

To test whether sampling the nasal epithelium of asthmatic donors would provide an improved method for preclinical evaluation, we obtained primary NEC's from asthmatic (*n* = 19) and non-asthmatic (*n* = 4) control donors by sampling the inferior turbinate with a Rhinoprobe™ curette, patient details and baseline data for mild, moderate and severe asthma patients are provided in Table 1. Irrespective of donor status, combined curettage of the right and left nasal turbinate produced similar total epithelial cell yields (up to 1 × 10^5^ cells, viability >70%). Attempts to culture cells obtained by curettage were initially hampered by high contamination rates. Subsequent cultures were therefore carried out in the presence of antibiotics, as described in the Materials and Methods section, to overcome this problem. Samples typically consisted of sheets of differentiated epithelium (Fig. 1A) with beating ciliated columnar epithelial cells readily discernible on microscopic examination (Fig. 1B). No evidence of contaminating cells was apparent from microscopic examination and gene expression analysis for the T cell marker CD3 was negative (data not shown). RT-PCR analysis of cells at the time of sampling showed that STAT6 mRNA expression was similar in nasal samples derived from either asthmatic (fold change, μ = 1.13 ± 0.61) or non-asthmatic (fold change, μ = 0.75 ± 0.12) donors (Fig. 1C). In contrast, CCL26 mRNA expression was significantly elevated in samples from asthmatic (fold change, μ = 31.6 ± 52.19) donors compared with the non-asthmatic (fold change, μ = 1.48 ± 1.3) controls (Fig. 1D). In addition, although not significant, a trend suggesting elevated expression of MUC5AC mRNA was also noted in the asthmatic group (fold change, μ = 169.37 ± 461.9) compared with the non-asthmatic (fold change, μ = 1.45 ±1.59) controls (Fig. 1E). No significant differences were noted between asthma patients with differing severities of disease, and no significant difference in expression levels was observed between CHI3L1 and PROM1 (data not shown). Therefore, NEC's derived from patients with bronchial asthma appear to exhibit phenotypic characteristics consistent with pro-asthmatic inflammatory events.

### Cultured primary NEC's exhibit rapid phenotypical changes

**Fig. 2:**
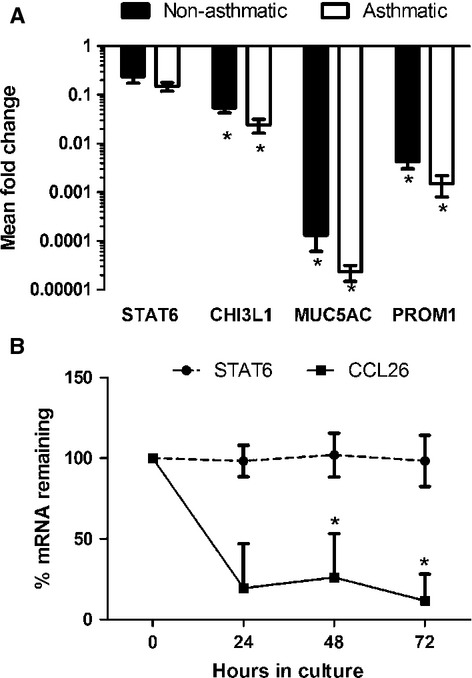
(**A**) Primary nasal epithelial cells from non-asthmatic (closed bars) or asthmatic (open bars) donors were cultured in epithelial growth medium for 30 days. Cells were harvested for RNA extraction to measure the relative expression of STAT6, CHI3L1, MUC5AC and PROM1 compared with expression at the time of nasal sampling (day 0). (**B**) Primary nasal epithelial cells from asthmatic donors were cultured in epithelial growth medium for 72 hrs. Cells were harvested for RNA extraction at 24, 48 or 72 hrs to measure the relative expression of STAT6 ( ) and CCL26 ( ) compared with expression at the time of nasal sampling (day 0). Values presented are the mean fold -change, relative to the mean δCt value for each group at day 0 ± SD. (A) Non-asthmatic: *n* = 4, asthmatic: *n* = 5. (B) Asthmatic: *n* = 3. Data were analysed by Student's *t*-(STAT6) or Mann–Whitney *U*-test (CHI3L1, MUC5AC, PROM1 and CCL26) and values differ from time of sampling (day 0): *P < 0.05.

Following harvesting, we attempted to culture primary NEC's briefly to enable treatment with STAT6-targeting siRNA. However, the limited primary cell yields precluded immediate siRNA testing; requiring extended (up to 30 days) culture in epithelial growth medium to obtain sufficient cells for further work. Comparative RT-PCR analysis of cells from mild asthmatic donors *versus* non-asthmatic controls (severe asthma patients were excluded because of relatively poorer cell yields) during this period showed that the epithelial differentiation markers MUC5AC, PROM1 and CHI3L1 mRNA expression were significantly reduced after 30 days in culture when compared with expression at the time of sampling (day 0) and this reduction was similar whether cells were obtained from asthmatic or non-asthmatic donors (Fig. 2A). Interestingly, however, no difference in STAT6 mRNA expression was noted (Fig. 2A). To investigate temporal CCL26 mRNA expression, three severe asthmatic patients whose NEC's had previously exhibited elevated CCL26 expression at time of sampling, were re-sampled and further gene expression analysis performed. Short culture (72 hrs) of NEC's from these donors revealed that whereas STAT6 expression was again shown to be relatively invariant, CCL26 mRNA expression was rapidly reduced during the first 24 hrs of culture and thereafter maintained at basal levels (Fig. 2B) highlighting the importance of the *in vivo* cytokine milieu in perpetuating the disease phenotype.

### Cultured primary NEC's are amenable to siRNA treatment

**Fig. 3:**
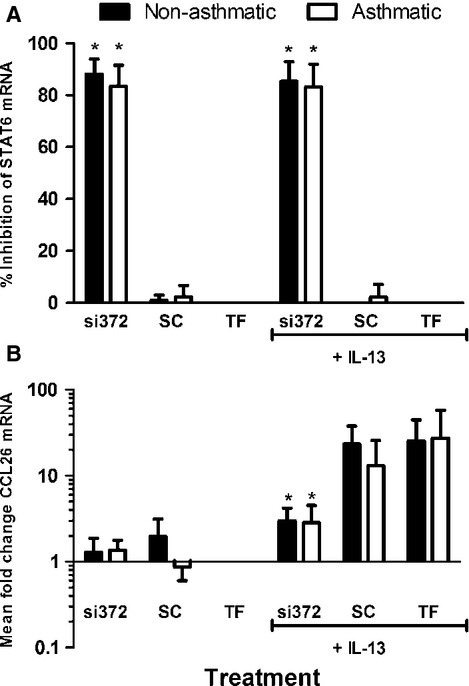
Primary nasal epithelial cells from non-asthmatic (closed bars) or asthmatic (open bars) donors were transfected with STAT6-targeting siRNA (si372), scrambled control siRNA (SC) or transfection reagent only (TF) either in the presence or absence of IL-13 (50 ng/ml). Cells were harvested for RNA extraction to measure the percentage inhibition of STAT6 (**A**) and relative expression of CCL26 (**B**) following 72-hr treatment. Values presented are percentage inhibition (STAT6) or mean fold change (CCL26), relative to the TF group ± SD (non-asthmatic controls: *n* = 4, asthmatic donors: *n* = 5). Data were analysed by Student's *t*-(STAT6) or Mann–Whitney *U*-test (CCL26) and values differ from TF: *P < 0.05.

As a consequence of the low cell yields obtained at the time of sampling and subsequent rapid phenotypic changes, we were unable to evaluate the effect of our siRNA in primary NEC's whilst still in their ‘*in vivo*’ differentiated state. We therefore sought to test our siRNA in the primary NEC's following culture on transwell membranes for up to 30 days. Cultured primary NEC's were treated with si372 as previously described, [12] and effects on STAT6 mRNA expression were measured by RT-PCR. Primary NEC's treated in this way exhibited reduced STAT6 expression and the extent of this inhibition was similar whether cells were derived from asthmatic or non-asthmatic donors. Furthermore, the extent of inhibition was not modulated by the addition of exogenous IL-13 (Fig. 3A). Notably, si372 treatment also attenuated the ability of IL-13 to up-regulate CCL26 mRNA expression in primary NEC cultures, again irrespective of donor asthma status (Fig. 3B). In addition to mRNA analysis, measurement of CCL26 protein expression in the basal culture medium by ELISA was also attempted. However, given the relatively low cell numbers, CCL26 protein concentrations were below the limit of detection by ELISA.

### Differentiated, polarized epithelial cells can be targeted by siRNA

**Fig. 4:**
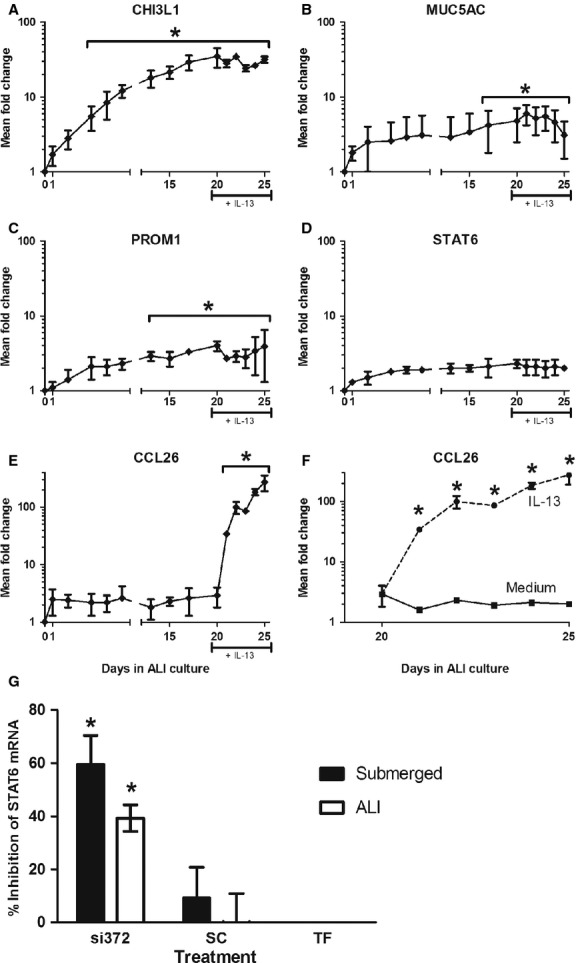
RPMI 2650 nasal epithelial cells were cultured as monolayers at an air–liquid interface in epithelial growth medium for 20 days, followed by further culture in epithelial growth medium containing 5 ng/ml IL-13 for 5 days. Cells were harvested for RNA extraction at days 1, 3, 6, 8, 10, 13, 15, 17 and daily from 20 to 25 to measure the relative mRNA expression of (**A**) CHI3L1, (**B**) MUC5AC, (**C**) PROM1, (**D**) STAT6 and (**E** and **F**) CCL26 by RT-PCR. (**G**) RPMI 2650 nasal epithelial cells grown under submerged (closed bars) or ALI (open bars) culture conditions were transfected with STAT6-targeting siRNA (si372), scrambled control siRNA (SC) or transfection reagent only (TF). Cells were harvested for RNA extraction to measure the percentage inhibition of STAT6 following 72-hr treatment. Values presented are mean fold change (A–F), relative to day 0, or percentage inhibition of STAT6 mRNA (G), relative to TF, ±SD (*n* = 3). Data were analysed by Student's *t*- (percentage inhibition) or Mann–Whitney *U*-test (fold -change) and values differ from day 0 or TF: *P < 0.05.

To evaluate whether siRNA treatment of polarized epithelial cells would be effective, we utilized RPMI 2650 cells grown under ALI conditions to promote the formation of differentiated polarized monolayers [23, 33, 34]. Tight monolayers formed after approximately 20 days of ALI culture and analysis of temporal gene expression showed that CHI3L1 (Fig. 4A), MUC5AC (Fig. 4B) and PROM1 (Fig. 4C) were up-regulated under these conditions, reaching a plateau on days 15–20 (fold-change day 20, μ = 35.0, 4.8, 4.0 respectively). In contrast, STAT6 (Fig. 4D) and CCL26 (Fig. 4E) mRNA expression was invariant over the period of ALI culture (mean fold -change ≤2.0). To confirm an intact STAT6 signalling pathway, monolayers were treated daily, between days 20 and 25, with IL-13 baso-laterally (5.0 ng/ml) and relevant gene expression measured over the ensuing 5 days. IL-13 treatment produced a marked up-regulation of CCL26 within 24 hrs which remained sustained for the period of IL-13 exposure (fold- change day 25, μ = 271.0 ± 82.8; Fig. 4E and F). In contrast, expression levels of CHI3L1, MUC5AC, PROM1 and STAT6 (Fig. 4A–D) were unaffected by IL-13 treatment.

Having determined that similar to primary cells, STAT6 gene expression was stable in RPMI 2650 cells, we addressed the feasibility of targeting STAT6 expression using si372. RPMI 2650 cells (submerged or ALI cultured) were transfected with si372 as previously described [12] with the exception that day 20 ALI-cultured cells were transfected for 24 hrs on their apical surface only to maintain ALI-induced differentiation (apical surface remained air exposed for the remaining 48 hrs of culture). Submerged or ALI-cultured RPMI 2650 cells treated with si372 exhibited reduced expression of STAT6 mRNA when compared with treatment with a non-targeting, scrambled control (SC) siRNA (Fig. 4G). Targeting of STAT6 mRNA in ALI-cultured cells was less efficient (% inhibition in submerged = 59.7 ± 10.7 *versus* 39.3 ± 4.9 in ALI cultures). To the best of our knowledge, this is a unique demonstration of specific siRNA activity in differentiated respiratory epithelial monolayer cells where siRNA has been delivered to the apical surface.

## Discussion

The complex nature of the inflammatory disorder underlying human asthma poses significant challenges to investigations of its molecular pathophysiology and the development of new therapeutics. As our candidate siRNA is intended to primarily target gene expression in airway epithelial cells of asthma patients [12, 13] we evaluated the activity of this compound in relevant cells, within the context of human disease. To avoid problems associated with animal models [14, 15] many studies utilize primary bronchial epithelial cells obtained through bronchoscopy or bronchial lavage. In this study we utilized a simpler, less invasive methodology based on curettage of nasal airway epithelium [21, 35, 36], an approach predicated on the accumulating evidence that allergic asthma is an example of an inflammatory disease affecting epithelial cells throughout the respiratory tract [37]. In addition to measuring target gene expression, we analysed gene expression associated with epithelial differentiation status and asthmatic disease in *ex vivo*-maintained primary NEC's.

In the absence of identifiable inflammatory cells, STAT6 mRNA expression was found to be invariant in primary NEC's from both asthmatic and non-asthmatic donors. In contrast, however, CCL26 expression was found to be significantly elevated in samples from asthmatic donors, suggesting nasal CCL26 has potential as a reliable epithelial biomarker that correlates with allergic bronchial asthma. Interestingly, previous studies with peripheral blood mononuclear cells [38] also suggested CCL26 as a potential IL-13-related biomarker in the context of asthma and our findings, in unmanipulated donor nasal epithelial cells, support this. The limited number of asthmatic donors used in this pilot study (*n* = 19), however, coupled with recent observations of non-IL-13 driven pathways in clinical disease [39], warrant further investigations into the reliability of nasal CCL26 expression as a biomarker of active human allergic airway disease.

One of the major aims of this study was to assess the activity of STAT6-targeting siRNA (si372) in epithelial cells derived from asthmatic individuals, with the presumption that this would be more indicative of the therapeutic capability of drug candidates. Although low cell yields associated with nasal curettage prevented analysis at the protein level, we have previously demonstrated that inhibition at the mRNA level directly correlates with elimination of detectable STAT6 protein expression in epithelial cells [12, 13], and RT-PCR analysis showed that NEC's from asthmatic donors were readily amenable to treatment with si372. Furthermore, this STAT6-suppressive treatment attenuated the ability of IL-13 to up-regulate pro-inflammatory CCL26 mRNA, whose expression has also been shown to directly correlate with CCL26 protein expression in lung epithelial cells [12]. Consistent with the invariant expression of STAT6, the level of si372-mediated inhibition of STAT6 mRNA expression was similar in cells from either donor group. This, coupled with evidence of elevated CCL26 expression in NEC's from asthmatic donors is consistent with IL-13-driven eotaxin expression in epithelial cells [40] and clinical observations of elevated CCL26 expression in bronchial asthma [25]. Despite the successful demonstration of siRNA activity in primary NEC's, it was notable that long-term culture of these cells was associated with a down-regulation of differentiation (CHI3L1, MUC5AC, PROM1) and disease (CCL26, MUC5AC)-associated markers, consistent with their removal from an *in vivo* pro-inflammatory milieu.

To enable characterization of the effectiveness of si372 in polarized epithelial cells, additional studies were performed with ALI-cultured RPMI 2650 monolayers, which we show adopt a gene expression profile consistent with differentiated airway epithelium, *i.e*. up-regulated mucin, prominin and chitinase family gene expression [21, 29, 41]. Interestingly, STAT6 mRNA expression, as in primary NEC's, was invariant during long-term culture of RPMI 2650 cells. Furthermore, ALI-cultured RPMI 2650 cells exhibited similar functional characteristics to both primary lung epithelial cells [12] and primary NEC's in that IL-13 responsiveness (CCL26 induction) was readily demonstrable. Furthermore, comparative studies in which ALI-differentiated RPMI 2650 cells were apically treated with si372 showed inhibition of STAT6 mRNA expression. This unique demonstration of siRNA activity when delivered to the apical surface of differentiated cultures suggests *in vivo* efficacy of si372 given that RPMI 2650 cells cultured under ALI conditions form polarized monolayers interconnected with tight junction proteins [23]. As polarized apical cells are generally resistant to lipid-based transfection agents, [42] this successful demonstration of target gene inhibition may be predicated on our use of polyethyleneimine (PEI) to transport siRNA into polarized epithelium. Indeed, the success of this approach is consistent with previous reports demonstrating that PEI-polyplexes are capable of transfecting polarized monolayers, whether of endothelial or epithelial origin [43]. Although speculative, *in vivo* targeting of asthmatic epithelium may actually be less challenging, given the characteristic loss of epithelial barrier integrity [44] and mucus metaplasia [45] associated with allergic asthma.

Although increases in STAT6 expression has been noted in the submucosa of respiratory epithelial biopsies from asthmatic patients, this is associated with the infiltration of IL-4^+^/CD3^+^ inflammatory cells [46, 47] and our observations of the relative invariance of STAT6 expression at the epithelial cell level combined with the attenuated expression of STAT6-dependent CCL26 post-siRNA treatment strengthen the rationale for targeting STAT6 in airway epithelial cells. Whether such a therapeutic would be targeted to bronchial or nasal epithelium would depend on siRNA-delivery capabilities (*e.g*. inhaled *vs*. topical administration) and future efficacy studies. However, it is interesting to note that nasal steroids have been shown to be of equal efficacy as an asthma treatment when compared with low doses of bronchial steroids [37], consistent with the concept of respiratory allergy as a single airway disease [20, 48]. Given our demonstration of the successful apical siRNA treatment of differentiated nasal epithelium and the relative accessibility of this route for patient drug administration, targeting asthma with siRNA-based drugs *via* the nasal route may be a productive first-line treatment strategy. In addition, as has previously been suggested [49], when combined with nasal curettage it may also provide a route through which drug efficacy could be readily tested as a part of the early drug-development process.

In conclusion, models of human nasal epithelium have potential in the *ex vivo* screening of candidate asthma therapeutics in that candidate siRNA activity is demonstrable in both primary cells and differentiated monolayer cultures. Given findings of elevated CCL26 expression in primary NEC's from asthmatic donors, curettage sampling has potential in both novel investigations of disease biomarker expression and early drug development. Both primary cells and ALI-differentiated monolayer cells displayed consistent target (STAT6) gene expression, suggesting that *in vivo* epithelial targeting of STAT6 may not be complicated by active disease. The ability of siRNA treatment to attenuate IL-13-induced CCL26 in primary NEC's from asthma patients further supports the therapeutic potential of this approach.
